# Sex trafficking and commercial sex: outcomes in suicide ideation and economic strain among young women

**DOI:** 10.3389/fpsyt.2026.1843240

**Published:** 2026-07-17

**Authors:** David Okech, Liu Liu, Anna Cody, Eunmi Hwang, Frederick Konteh

**Affiliations:** 1 School of Social Work, University of Georgia, Athens, GA, United States; 2 University of Sierra Leone, Freetown, Sierra Leone

**Keywords:** commercial sex, economic strain, Senegal, sex trafficking, suicide ideation

## Abstract

**Introduction:**

Documenting the effects of sex trafficking on survivors’ health, psychological, and economic needs is pivotal for designing responsive and comprehensive interventions. Although women engaged in commercial sex may be at heightened risk of experiencing sex trafficking, there is limited evidence about how outcomes may differ among women who may be survivors of sex trafficking and those who have not experienced sex trafficking. We examine differences in suicidality, economic strain, food security, and self-efficacy by sex trafficking classification and tested whether economic strain, food security, and self-efficacy were associated with suicidality beyond trafficking classification.

**Methods:**

We analyzed endline survey data from women engaged in commercial sex in the artisanal and small-scale gold mining area of Kédougou, Senegal, recruited using link-tracing sampling, and estimated survey-weighted descriptive and regression models of suicidality severity and any suicidality in relation to sex trafficking classification, economic strain, food security, self-efficacy, and age. We also constructed four trafficking experience domains and a cumulative domain count to examine heterogeneity beyond a binary classification.

**Results:**

In the full analytic sample (*N* = 842), 450 respondents (53.4%) were classified as individuals who experienced sex trafficking. In survey-weighted multivariable models adjusting for age, economic strain, food security, and self-efficacy, sex trafficking classification was not independently associated with suicidality. Greater economic strain was associated with higher suicidality severity, whereas higher self-efficacy was associated with lower severity. In the full sample, food security showed a trend toward lower suicidality (*p* <.10). Findings were consistent when suicidality was modeled as a binary outcome. Domain-based trafficking indicators differed markedly by trafficking classification, but no single domain uniquely predicted suicidality after adjustment; restriction of freedom of movement showed the most consistent trend, and cumulative domain exposure showed a modest dose–response association with suicidality severity.

**Discussion:**

Suicidality among women engaged in commercial sex was more strongly associated with economic strain and self-efficacy than with binary sex trafficking classification. Findings support integrated responses that address material hardship and strengthen coping capacity, while screening approaches may benefit from capturing cumulative trafficking-related harms and indicators of restricted freedom. The distinction between commercial sex and sex trafficking is complex and blurred, and interventions should not exclude those not identified as sex trafficking victims because they may present similar needs to those identified as victims of sex trafficking.

## 1 Introduction

**Figure 1:**
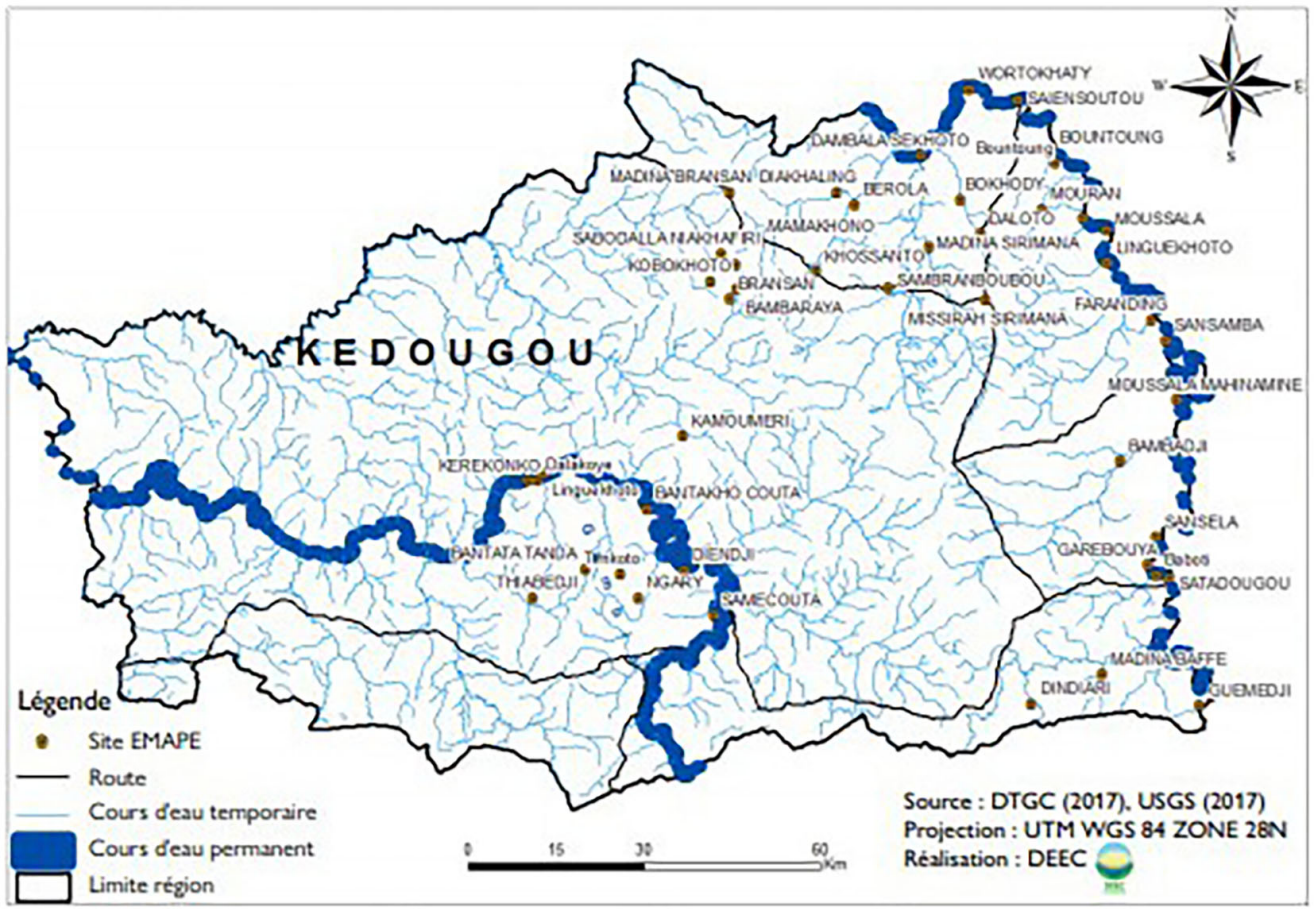
Map of artisanal and small-scale gold mining sites in Kedougou, Senegal.

Human trafficking for the purposes of sexual exploitation affects an estimated 6.3 million individuals, 4 out of 5 of whom are women and girls, across the globe (1). In southeastern Senegal, West Africa, women and girls are sexually exploited in the artisanal and small-scale gold mining (ASGM) sector (2, 3), with close to half of the women engaged in commercial sex in the region identified as individuals who experienced sex trafficking (3). In addition to the high prevalence of sex trafficking among adult women engaged in commercial sex, previous research found that children as young as eleven had experienced sex trafficking in Kédougou (2, 3). This exploitation is driven by socioeconomic conditions and the demand for cheap labor and sexual services (2). Gold mining regions, especially Kédougou, have become hotspots for sex trafficking (**Figure 1**). The gold rush in these areas has attracted a large influx of miners and associated workers, creating a lucrative environment for traffickers (4). Victims of sex trafficking are exposed to multiple forms of trauma - sexual violence, physical assault, coercion, and psychological manipulation (5–7). Significant and lasting impacts on mental and physical health have been associated with human trafficking; among people who experience sex trafficking, depression, anxiety, Post Traumatic Stress Disorder (PTSD), and complex PTSD are common (7–10). Sex trafficking survivors have also reported relational difficulties and disassociation (8).

## 2 Literature review

### 2.1 Context of commercial sex in Senegal

Senegal has regulated commercial sex since 1969 to reduce the incidence of sexually transmitted diseases (STIs), especially Human Immunodeficiency Virus (HIV) (11). Engagement in commercial sex is legal for adults over 21 years old who have registered with the health ministry (11–14). One key feature of the registration program is that women who are registered with a health card attend a monthly visit to a health post. However, some research indicates that the majority of women engaged in commercial sex are not registered with the health department, so may have less contact with health workers or other authorities, and may also experience discrimination due to language and cultural barriers (11, 12). Recent estimates from a national survey of sexual and reproductive health in Senegal indicate that 80% of women engaged in commercial sex are not registered (12). Further, some studies indicate that registration may be protective of physical health but may negatively affect subjective well-being (11).

For example, a study on the effects of sex work regulation on the health and well‐being of sex workers in Senegal found that women engaged in commercial sex in Senegal who were registered were 11 percentage points less likely to report being sick or having an injury but were 8 percentage points more likely to report being unhappy with life in general compared to women engaged in commercial sex who were not registered. Collectively, scholars suggest that stigma and social barriers may be drivers for women not registering with the health department and for negative effects on mental health when they do register with the health department (11, 12, 15). Current scholarship suggests that modernizing the registration system, such as using digital health cards rather than physical health cards and including psychosocial support as part of regular health visits, may increase the number of women who register with the health department and also reduce potential negative impacts from registration (11, 12).

### 2.2 Context of sex trafficking in Senegal

Research about sex trafficking in Senegal is limited. Typically, stakeholders rely on administrative records, such as law enforcement and court records, to understand the scope and impact of sex trafficking. Recent research, which we conducted in 2024, estimated that nearly 50% of women engaged in commercial sex in the Kédougou region had experienced sex trafficking within the 12 months prior to the survey (3). Among those surveyed, the majority who experienced sex trafficking were from Nigeria (82%) and Senegal (10%). Adverse childhood experiences significantly heightened the risk of experiencing sex trafficking in adulthood. When asked about childhood experiences, 60% reported facing physical abuse, 15% experienced sexual violence, and 26% were exposed to alcohol (3). Additionally, poor living conditions prior to experiencing sex trafficking, such as lack of food (28%) and overcrowded housing (21%), were identified as drivers for women seeking risky income opportunities, increasing potential for manipulation by traffickers (3).

Descriptive findings explored multiple forms of material hardship among women engaged in commercial sex and women who had experienced sex trafficking, including insufficient food, lack of safe housing, and limited access to necessities, and suggested that each additional form of deprivation increases the likelihood of suicidality (3). Our previous descriptive results from Senegal are consistent with the scant literature that explores sex trafficking prevalence and incidents among women engaged in commercial sex globally. Research conducted in Bangladesh, India, and Nicaragua found that experiences of sex trafficking, specifically entering commercial sex through sex trafficking situations, are common, with estimates ranging from 24% to 75% of individuals engaged in commercial sex (16–18). More research is needed to understand the variability and complexities in sex trafficking risk and for women engaged in commercial sex.

### 2.3 Effects of sex trafficking

Sex trafficking is associated with significant and lasting impacts on physical, psychological, and economic well-being. Common mental health outcomes among survivors include depression, anxiety, PTSD, complex PTSD, and suicidal ideation (19–21). Sex trafficking also affects the coping and self-efficacy of victims; however, these effects can be mitigated through various interventions and support for victims (22, 23). Specifically, one study (23) found that community reintegration indirectly influenced PTSD through its effect on perceived social support. Survivors who reported more difficulty reintegrating into the community perceived less social support than those who reported easier community reintegration, and trafficking survivors who perceived less social support indicated more PTSD symptoms. Survivors with more PTSD symptoms tend to report using more dysfunctional coping mechanisms (23), and the coping can be demonstrated in alternative ways, such as refusing treatment (22).

While the psychological toll of trafficking-related violence is well established, others have turned greater attention to the structural conditions that shape both vulnerability and recovery (24). Many survivors continue to face persistent economic hardship after leaving exploitative environments - housing instability, food insecurity, and limited social support remain common realities (25). Such conditions may intensify psychological distress and complicate long-term recovery. An appreciation of the interaction between health, psychological, and economic effects of sex trafficking on women is important in designing effective, comprehensive interventions (25–29). As Doyydaitis (30) noted, sex trafficking survivors may experience different combinations of physical, economic, sexual, and psychological exploitation.

### 2.4 The present study

The present study extends and builds on our previous multi-level intervention research study in Kédougou Senegal, whose goal was to evaluate changes in sex trafficking prevalence, perceptions, and policy (2, 3). Overall, the study included three primary phases: 1) baseline prevalence study conducted in 2021, 2) program implementation, between 2021 and 2024, focused on protection, survivor care, and reintegration and prosecution, and 3) an endline prevalence study conducted in 2024 (2, 3). The intervention program emphasized community-based prevention (e.g., vigilance committees and awareness campaigns), survivor protection through trauma-informed services, and reintegration support, as well as strengthened prosecution through legal training and interagency coordination—resulting in increased identification of survivors and successful convictions (3). Repatriation and reintegration services were particularly important for survivor well-being. Overall, the intervention demonstrated that coordinated, survivor-centered, and multi-sector efforts can significantly improve identification, support, and justice outcomes, while underscoring the importance of strengthening prevention efforts in countries of origin like Nigeria (3). Results from our collective previous research and programming initiatives were used to develop evidence-driven recommendations for enhanced prevention and response efforts (2, 3).

The specific goal of this present study is to analyze and quantify health and economic differences between women who had experienced and those who did not experience sex trafficking within the sample of commercial sex workers in the ASGM of Kédougou, Senegal. The study answers the following three research questions:

1. Among women engaged in commercial sex, do suicidality, self-efficacy, economic strain, and food security differ between respondents classified as sex trafficking versus not classified?
2. After adjusting for age, economic strain, food security, and self-efficacy, is sex trafficking classification associated with suicidality severity and any suicidality?
3. Do the domains of sex trafficking and cumulative domain exposure relate to suicidality outcomes beyond economic strain, food security, and self-efficacy, including within the sex trafficking subsample?

## 3 Methods

### 3.1 Design and participants

#### 3.1.1 Link-tracing sampling

Data analyzed for this study are drawn from the endline survey collected in 2024 from women engaged in commercial sex in the ASGM area of Kédougou, Senegal (3). The endline survey employed Link-Tracing Sampling (LTS), a respondent-driven sampling and recruitment approach. LTS was selected for this study because it is particularly effective in research that engages with “hard-to-reach” populations (3). LTS uses a referral-chain process (similar to snowball sampling) to identify people who may be eligible to participate in the study. The process starts with a small group of “seeds,” people initially recruited into a study, who are asked to refer people they know to join the study using a study referral coupon. Each new recruit is then asked to refer some people that they know, which constitutes a recruitment “wave”. In preliminary analysis during the endline study, Volz-Heckathorn weights were applied to support population inference within the recruited networks (31).

#### 3.1.2 Ethics and data collection process

The study was approved by IRB review boards, including the Comité National d’éthique pour la Recherche en Santé (CNERS) in Dakar, Senegal, as well as a University in the Southeastern United States (3). Prior to data collection activities, the US-based research team and the research partner in Senegal collaboratively trained data collectors in research ethics. Our safety and ethics protocol aligned with best practices for interviewing women who have experienced sex trafficking, emphasizing respondent safety, privacy, and choice (32). Prior to any data collection activities, all data collectors and team members of our Senegalese-based research partner were required to attend a trauma-informed training that included: 1) a basic understanding of trauma impacts on emotional regulation and behaviors, 2) common signs that someone is being actively triggered (e.g., tears welling up) and how to respond, 3) stressing that consent is a process and respondents have the right to stop an interview at any time, 4) steps for minimizing personal bias and judgment. Interviews were conducted in private spaces within local health centers, where women who are engaged in commercial sex regularly attend health check-ups. The health centers were selected as interview sites to provide an additional layer of support to respondents. Should they experience distress during an interview or indicate a need to follow up, data collectors were trained to link them to community health workers present in the health facilities. In addition, we linked survivors to services, such as shelter support, through our intervention program partners.

Women who were between 18–30 years old, who had been engaged in commercial sex within the 12 months prior to data collection, resided in the target departments (Kédougou and Saraya) within the Kédougou region, and were proficient in English, French, or the local language were eligible to participate in the survey. Respondents were compensated with 5,000 CFA (approximately $10 USD) for their participation in an interview and an additional 5,000 CFA (approximately $10 USD) for each successful referral (3). Through outreach to the target population, the research team recruited initial seeds (51 in Kédougou and 49 in Saraya) and then provided respondents with 2 referral coupons to give to women they knew. Consistent with LTS methodology, teams collected data at community health centers within the study districts. Survey interviews were conducted in a private space within the local health centers. Data collection activities were monitored by the research partner and study coordinators, who were Senegalese-based staff of the US University research center conducting the study (3).

The total number of women surveyed for the endline study was 850 (3). However, the eligible analytic dataset included *N* = 842 respondents with valid weights and a sex trafficking classification (3). Outcome-specific analytic sample sizes varied due to item nonresponse. Models that simultaneously included economic strain, food security, self-efficacy, and age were estimated on complete cases (*n* = 824 in the full sample).

### 3.2 Instruments and measures

#### 3.2.1 Sex trafficking classification

Sex trafficking is defined under U.S. federal law as the recruitment, harboring, transportation, provision, or obtaining of a person for the purposes of a commercial sex act in which the commercial sex act is induced by force, fraud, or coercion, or in which the person induced is under 18 (22 U.S.C. § 7102). In this federal definition, force, fraud, or coercion is not required when the person induced is under 18; however, the present study sample included adults aged 18–30. Sex trafficking status in this study was represented by a binary indicator provided in the analytic dataset (1 = classified as sex trafficking, 0 = not classified). As described in the endline report (3), respondents were classified as experiencing sex trafficking if they endorsed at least one sex trafficking indicator in the 12 months prior to the survey across four exploitation categories assessed in the survey: exploitative recruitment, employment practices and debt, coercion and control, and loss of freedom of movement.

These categories were operationalized through behaviorally specific survey items rather than requiring respondents to define abstract legal concepts such as force, fraud, or coercion. Enumerators were trained in the meaning of these concepts and in administering and consistently explaining the relevant items to participants during data collection. Representative example indicator items are summarized in **Table 1**. In the analytic sample, 450 of 842 respondents (53.4%) were classified as sex trafficking. In the complete-case sample used for multivariable models (*N* = 824), 439 respondents (53.3%) were classified as sex trafficking.

**Table 1:** Measures, scoring, and reliability.

Construct	Items and response options	Scoring	Reliability in this sample	Validity or conceptual basis
Sex trafficking classification	Binary classification derived from past-12-month exploitation indicators assessed across four categories (exploitative recruitment; employment practices and debt; coercion and control; loss of freedom of movement). Example indicators included forced/coerced commercial sex and restricted movement or surveillance.	1 = classified as sex trafficking; 0 = not classified	Not applicable	Endline report operational definition (4), consistent with a force–fraud–coercion framework and 22 U.S.C. § 7102
Suicidality (USSIS)	4 items; 7-point frequency scale	Mean of items; any suicidality = any item ≥ 4	Cronbach’s = 0.88; ordinal = 0.96	19, 20
Self-efficacy	6 items; 5-point agreement scale (1 = strongly agree to 5 = strongly disagree)	Reverse-coded as needed; mean of items	Cronbach’s = 0.92; ordinal = 0.94	Adapted from General Self-Efficacy content (21)
Economic strain	8 hardship indicators (living-condition hardship checklist)	Deprivation count index (sum; range 0–8), higher = greater strain	Not applicable	Study-developed index
Food security	1 item; 5-point agreement scale (1 = strongly agree to 5 = strongly disagree)	Reverse-coded so higher = more secure	Not applicable	Study item
Age	Self-reported in years	Continuous	Not applicable	Study item

USSIS, Ultra-Short Suicidal Ideation Scale. Ordinal α was computed using polychoric correlations as a sensitivity check for ordinal response formats. Additional sex trafficking indicators included deceptive recruitment about work and withheld wages or debt-related constraints.

##### 3.2.1.1 Decision rule clarification

The binary sex trafficking indicator followed the endline report operational definition described above. The four trafficking experience domain indicators constructed for this manuscript were designed to summarize broader patterns of exploitation experiences aligned with the same categories and to examine heterogeneity beyond a binary classification. Because these domains capture any endorsement of relevant experiences, and some items may reflect vulnerability or exploitation that does not meet the endline report’s classification threshold, domain endorsement can occur among respondents not classified as sex trafficking.

#### 3.2.2 Suicidality

Suicidality was assessed using the Ultra-Short Suicidal Ideation Scale (USSIS), a four-item measure of current suicidal thinking and intent (33). Items were rated on a 7-point frequency scale and were averaged to create a continuous suicidality severity score, with higher values indicating greater suicidality. For binary models, any suicidality was defined as reporting at least one item at response level 4 or higher. Internal consistency in the present sample was high (Cronbach’s alpha = 0.88; ordinal alpha = 0.96). Prior work has reported evidence for the validity and measurement equivalence of the USSIS (34).

#### 3.2.3 Self-efficacy

Self-efficacy was assessed with six Likert-type items capturing perceived problem-solving and coping capacity, rated on a 5-point agreement scale (1 = strongly agree to 5 = strongly disagree). Items were reverse-coded so that higher values indicated higher self-efficacy, and a mean score was computed across items. Internal consistency was high (Cronbach’s alpha = 0.92; ordinal alpha = 0.94). The self-efficacy construct has established reliability and validity evidence across cultural contexts (31).

#### 3.2.4 Economic strain

Economic strain was operationalized as a material deprivation count index based on eight living condition hardship indicators assessed via a multiple-response checklist referring to conditions experienced prior to engaging in commercial sex. The eight hardships included: insufficient food; living and sleeping in overcrowded rooms; sleeping in dangerous conditions; overexposure to sun or rain; poor basic hygiene; inadequate drinking water; having no clean clothes; and having nowhere to sleep or sleeping on the floor. Each hardship was coded as endorsed (1) versus not endorsed (0), and items were summed to yield a deprivation count (range 0–8), with higher scores indicating greater economic strain. “Don’t know” and “refused” responses were treated as missing.

#### 3.2.5 Food security

Food security was measured with a single item assessing perceived ability to obtain enough food when hungry, rated on a 5-point agreement scale (1 = strongly agree to 5 = strongly disagree). Responses were reverse-coded so that higher values indicated greater food security.

#### 3.2.6 Covariate

Age was self-reported in years and included as a minimal adjustment covariate because, even within the 18–30-year sampling frame, age may be associated with duration of exposure to commercial sex and exploitation, economic circumstances, and baseline mental health risk.

#### 3.2.7 Trafficking experience domain indicators

To examine heterogeneity in sex trafficking experience beyond a binary classification, we constructed four trafficking experience domain indicators aligned with the exploitation categories described in the endline report, and with force-, fraud-, and coercion-based trafficking identification and prevalence measurement frameworks. The four domains were: (1) recruitment and entry mechanisms, (2) employment and debt-related exploitation, (3) coercion and control, and (4) mobility restriction and surveillance. These domains correspond to behaviorally specific indicators commonly used to identify trafficking-related exploitation, including deceptive or forced recruitment, debt or wage-related exploitation, threats or coercive control, restriction of movement, surveillance, and confiscation of identity documents or personal property (30, 35, 36). Each domain was coded 1 if the respondent endorsed any item in that domain and coded 0 if at least one domain item was observed and none were endorsed. A domain was coded as missing only if all items in that domain were missing. A domain count variable (range 0–4) was defined as the number of domains endorsed and treated as a cumulative exposure measure.

### 3.3 Missing data and sensitivity analysis

Special missing codes were recoded as missing. Outcome-specific analytic sample sizes ranged from *n* = 826 to *n* = 842 due to item nonresponse (suicidality severity *n* = 840, any suicidality *n* = 840, self-efficacy *n* = 842, economic strain *n* = 826, food security *n* = 841, and assets *n* = 842). Suicidality and self-efficacy scores were computed using available items when at least one item response was observed. Regression models used complete-case estimation for the outcome and predictors included in each model (multivariable models *n* = 824).

Item diagnostics identified two trafficking items with high missingness consistent with skip patterns. As a robustness check, we reconstructed the employment and debt domain, excluding the item on acts performed to repay money, and reconstructed the coercion and control domain, excluding the item on reprisals. We then re-estimated domain prevalence and regression models. Results were substantively unchanged.

### 3.4 Statistical analysis

All analyses were conducted in R (version 4.3.1). Survey-weighted analyses used the survey package (Lumley, 2004) with RDS weights and design-based robust standard errors (single-stage design with ids = ~1). We first computed weighted descriptive statistics (means, standard deviations, and prevalences) for suicidality, self-efficacy, and economic indicators by sex trafficking classification. We then estimated primary multivariable models with suicidality as the outcome: (a) survey-weighted linear regression for suicidality severity (continuous mean score) and (b) survey-weighted logistic regression (quasibinomial) for any suicidality, with predictors including sex trafficking classification, economic strain, food security, self-efficacy, and age.

To examine trafficking experiences heterogeneity, we constructed four trafficking experience domain indicators reflecting recruitment and entry mechanisms, employment or debt-related exploitation, coercion and control, and restriction of freedom of movement. We estimated: (a) weighted domain prevalence overall and by sex trafficking classification, (b) domain-only models predicting suicidality outcomes from the four domain indicators and age, and (c) fully adjusted domain models that additionally controlled for economic strain, food security, and self-efficacy. We repeated the fully adjusted domain models within the subset of respondents classified as sex trafficking to assess variation in suicidality within the trafficking group. We also fit dose–response models using the trafficking domain count (0–4) as a cumulative exposure measure. Wald-type 95% confidence intervals were computed using robust standard errors.

## 4 Results

### 4.1 Scale reliability

Internal consistency was high for both multi-item scales. The 4-item suicidality scale demonstrated strong reliability (Cronbach’s alpha = 0.88; ordinal alpha = 0.96), and the 6-item self-efficacy scale also demonstrated strong reliability (Cronbach’s alpha = 0.92; ordinal alpha = 0.94). Ordinal alphas were computed from polychoric correlations as a sensitivity check, given the ordinal response format.

### 4.2 Weighted descriptives by sex trafficking classification

Weighted descriptive comparisons indicated modest differences between respondents classified as sex trafficking versus those not classified. In the analytic sample (*N* = 842), 450 respondents were classified as sex trafficking, and 392 were not classified. As shown in **Table 2**, the sex trafficking group reported slightly higher suicidality severity (weighted M = 1.56 vs 1.43) and slightly higher economic strain (weighted M = 1.54 vs 1.40), whereas self-efficacy and food security were similar across groups. **Table 2** summarizes weighted means and standard deviations for suicidality, self-efficacy, economic strain, food security, and household assets.

**Table 2:** Weighted descriptives by sex trafficking classification.

Outcome	Sex trafficking *n*	Sex trafficking M	Sex trafficking SD	Not classified *n*	Not classified M	Not classified SD
Suicidality severity (mean)	449	1.56	1.09	391	1.43	0.88
Self-efficacy (mean)	450	3.78	0.95	392	3.83	0.91
Economic strain (deprivation count)	441	1.54	1.42	385	1.40	1.20
Food security (higher = more secure)	449	4.08	1.06	392	4.11	1.09
Household assets count	450	3.02	1.78	392	2.84	1.71

Descriptives are survey-weighted using RDS weights. Ns vary by outcome due to item nonresponse.

### 4.3 Primary multivariable models of suicidality

In survey-weighted multivariable models adjusting for age, economic strain, food security, and self-efficacy, greater economic strain was associated with higher suicidality severity (b = 0.078, *p* = 0.017), whereas higher self-efficacy was associated with lower suicidality severity (b = -0.183, *p* <.001). In the full sample, food security showed a trend toward lower suicidality severity (b = -0.071, *p* = 0.079). Sex trafficking classification was not significantly associated with suicidality severity after accounting for these covariates (b = 0.111, *p* = 0.129) (see **Table 3**). These multivariable models were estimated using the complete-case analytic sample (*N* = 824).

**Table 3:** Primary survey-weighted models predicting suicidality.

Panel A. Linear model predicting suicidality severity				
Predictor	b	SE	95% CI	*p*
Sex trafficking status (1 vs 0)	0.111	0.073	[-0.032, 0.254]	0.129
Economic strain (deprivation count)	0.078	0.033	[0.014, 0.143]	0.017
Food security (higher = more secure)	-0.071	0.040	[-0.150, 0.008]	0.079
Self-efficacy (higher = greater efficacy)	-0.183	0.054	[-0.289, -0.078]	< .001
Age (years)	0.010	0.012	[-0.015, 0.034]	0.425
Panel B. Logistic model predicting any suicidality				
Predictor	OR	95% CI low	95% CI high	*p*
Sex trafficking status (1 vs 0)	1.14	0.77	1.70	0.503
Economic strain (deprivation count)	1.22	1.06	1.41	0.006
Food security (higher = more secure)	0.87	0.73	1.02	0.094
Self-efficacy (higher = greater efficacy)	0.69	0.56	0.85	< .001
Age (years)	1.00	0.94	1.07	0.892

Models adjust for age. Economic strain, food security, and self-efficacy are entered simultaneously with sex trafficking classification. Full-sample complete-case *n* = 824. OR, odds ratio.

Results were consistent when suicidality was modeled as a binary outcome. Economic strain was associated with higher odds of any suicidality (OR = 1.22, 95% CI 1.06–1.41, *p* = 0.006), while self-efficacy was protective (OR = 0.69, 95% CI 0.56–0.85, *p* <.001). Food security showed a trend toward lower odds of any suicidality (OR = 0.87, 95% CI 0.73–1.02, *p* = 0.094), and sex trafficking classification was not significant (OR = 1.14, 95% CI 0.77–1.70, *p* = 0.503) (see **Table 3**).

### 4.4 Trafficking experience domains

Using the reduced domain definitions that excluded two skip-pattern items, weighted domain prevalence in the full sample was 51.4% for recruitment and entry mechanisms, 41.1% for employment and debt or financial exploitation, 23.8% for coercion and control, and 21.9% for mobility restriction and surveillance (see **Table 4**). Domain prevalence differed sharply by sex trafficking classification. Among respondents not classified as sex trafficking, coercion and control (3.6%) and mobility restriction (2.6%) were rare, whereas among respondents classified as sex trafficking, these domains were common (43.3% and 40.5%, respectively). The mean number of domains endorsed was 0.421 among respondents not classified as sex trafficking versus 2.31 among respondents classified as sex trafficking. Any domain endorsement was 28.5% versus 92.7%, respectively (see **Table 4**).

**Table 4:** Trafficking experience domains. weighted prevalence and overlap.

Domain	Overall (weighted %)	Not classified (weighted %)	Sex trafficking (weighted %)
Recruitment and entry mechanisms	51.4	20.1	81.6
Employment and debt or financial exploitation	41.1	15.8	65.6
Coercion and control	23.8	3.6	43.3
Mobility restriction and surveillance	21.9	2.6	40.5

Based on reduced domain definitions excluding two skip-pattern items. Mean domain count (0-4) was 0.421 for not classified and 2.310 for sex trafficking. Any domain endorsement was 28.5% for not classified and 92.7% for sex trafficking.

In adjusted domain models that included age, economic strain, food security, and self-efficacy, none of the four trafficking domains showed statistically significant independent association with suicidality severity or any suicidality (see **Table 5**). Mobility restrictions showed the strongest trend toward increased odds of any suicidality in the full sample (OR = 1.69, 95% CI [0.864, 3.29], *p* = .125). Economic strain and self-efficacy remained consistently associated with suicidality outcomes in these models.

**Table 5:** Strategy 1. Adjusted trafficking domain models (Full sample).

Panel A. Linear model predicting suicidality severity				
Predictor	b	SE	95% CI	*p*
Recruitment and entry mechanisms	0.088	0.086	[-0.080, 0.256]	0.305
Employment and debt or financial exploitation	-0.015	0.100	[-0.211, 0.181]	0.880
Coercion and control	0.203	0.136	[-0.064, 0.471]	0.136
Mobility restriction and surveillance	-0.007	0.155	[-0.312, 0.298]	0.964
Age (years)	0.009	0.012	[-0.015, 0.033]	0.458
Economic strain (deprivation count)	0.074	0.032	[0.010, 0.138]	0.023
Food security (higher = more secure)	-0.072	0.041	[-0.151, 0.008]	0.078
Self-efficacy (higher = greater efficacy)	-0.178	0.053	[-0.282, -0.074]	< .001
Panel B. Logistic model predicting any suicidality (ORs)				
Predictor	OR	95% CI low	95% CI high	*p*
Recruitment and entry mechanisms	1.01	0.62	1.65	0.954
Employment and debt or financial exploitation	0.94	0.52	1.70	0.848
Coercion and control	0.88	0.45	1.70	0.700
Mobility restriction and surveillance	1.69	0.86	3.29	0.125
Age (years)	1.01	0.95	1.08	0.802
Economic strain (deprivation count)	1.22	1.06	1.41	0.005
Food security (higher = more secure)	0.86	0.73	1.03	0.094
Self-efficacy (higher = greater efficacy)	0.69	0.55	0.85	< .001

Adjusted models include all four trafficking domains, age, economic strain, food security, and self-efficacy. Full-sample complete-case *n* = 824. Domain indicators are binary (1 = any endorsement).

Among respondents classified as individuals who experienced sex trafficking, adjusted models similarly indicated strong associations of higher economic strain with higher odds of any suicidality (OR = 1.35, *p* <.001) and higher self-efficacy with lower odds of suicidality (OR = 0.60, *p* <.001), while domain indicators did not show statistically significant unique associations (see **Table 6**). Results were substantively unchanged in robustness checks that reconstructed domains excluding the two skip-pattern items.

**Table 6:** Adjusted trafficking domain models among respondents classified as sex trafficking.

Panel A. Linear model predicting suicidality severity				
Predictor	b	SE	95% CI	*p*
Recruitment and entry mechanisms	0.042	0.134	[-0.220, 0.304]	0.756
Employment and debt or financial exploitation	-0.015	0.119	[-0.248, 0.218]	0.902
Coercion and control	0.235	0.145	[-0.049, 0.518]	0.106
Mobility restriction and surveillance	0.028	0.170	[-0.305, 0.362]	0.868
Age (years)	0.014	0.016	[-0.017, 0.046]	0.374
Economic strain (deprivation count)	0.121	0.045	[0.032, 0.210]	0.008
Food security (higher = more secure)	-0.044	0.059	[-0.160, 0.071]	0.452
Self-efficacy (higher = greater efficacy)	-0.251	0.077	[-0.401, -0.100]	0.001
Panel B. Logistic model predicting any suicidality (ORs)				
Predictor	OR	95% CI low	95% CI high	*p*
Recruitment and entry mechanisms	1.02	0.48	2.19	0.956
Employment and debt or financial exploitation	0.98	0.47	2.04	0.953
Coercion and control	0.85	0.41	1.77	0.670
Mobility restriction and surveillance	1.78	0.84	3.77	0.130
Age (years)	1.01	0.93	1.10	0.800
Economic strain (deprivation count)	1.35	1.13	1.61	< .001
Food security (higher = more secure)	0.89	0.70	1.13	0.339
Self-efficacy (higher = greater efficacy)	0.60	0.45	0.81	< .001

Models are restricted to respondents classified as sex trafficking (*n* = 450 total; complete-case *n* varies by outcome and predictors) and are survey-weighted. Predictors are entered simultaneously.

### 4.5 Dose-response model using domain count

A domain-count model indicated a modest dose–response association with suicidality severity. In the adjusted survey-weighted linear model controlling age, economic strain, food security, and self-efficacy, each additional trafficking domain endorsed was associated with higher suicidality severity (b = 0.0628 per domain, *p* = .019) (see **Table 7**). In the adjusted logistic model predicting any suicidality, the domain count was not statistically significant (b = 0.0757, *p* = .250; OR = 1.08 per additional domain), whereas economic strain remained associated with higher odds of any suicidality (b = 0.1969, *p* = .006; OR = 1.22) and self-efficacy remained protective (b = -0.3649, *p* <.001; OR = 0.69) (see **Table 7**).

**Table 7:** Dose-response models using trafficking domain count.

Panel A. Linear model predicting suicidality severity (adjusted)				
Predictor	b	SE	95% CI	*p*
Trafficking domain count (0-4)	0.063	0.027	[0.011, 0.115]	0.019
Age (years)	0.010	0.012	[-0.014, 0.034]	0.426
Economic strain (deprivation count)	0.076	0.033	[0.012, 0.140]	0.021
Food security (higher = more secure)	-0.072	0.040	[-0.151, 0.007]	0.074
Self-efficacy (higher = greater efficacy)	-0.178	0.053	[-0.282, -0.074]	< .001
Panel B. Logistic model predicting any suicidality (adjusted)				
Predictor	OR	95% CI low	95% CI high	*p*
Trafficking domain count (0-4)	1.08	0.95	1.23	0.250
Age (years)	1.00	0.94	1.07	0.892
Economic strain (deprivation count)	1.22	1.06	1.40	0.006
Food security (higher = more secure)	0.86	0.73	1.02	0.086
Self-efficacy (higher = greater efficacy)	0.69	0.56	0.86	< .001

Domain count ranges from 0 to 4 and reflects the number of trafficking domains endorsed. Adjusted models include age, economic strain, food security, and self-efficacy. Full-sample complete-case *n* = 824.

## 5 Discussion

### 5.1 Implications for practice

The finding that greater economic strain was associated with higher suicidality is consistent with the general strain theory (GST), which suggests that economic strain can lead to intense psychological distress, hopelessness, and reduced “lifetime utility,” thus increasing suicide risk (37). These frameworks emphasize that cumulative financial pressures—job loss, debt, and food security—set off intense emotional crises that can be fatal. Living under sustained economic strain, as was the case with our target population, in addition to the awful working conditions, can lead to suicidal ideation. Given that economic insecurity is what pushed most of these women into sex work (3), continuing to live under economic insecurity is a double burden for them if their work cannot address their economic needs. A trauma-focused intervention is therefore necessary to respond to the mental health as well as the economic needs of this population.

To address the concern that a binary trafficking indicator may obscure heterogeneity in exploitation experiences, we decomposed trafficking experiences into four domains reflecting recruitment and entry mechanisms, employment or debt-related exploitation, coercion and control, and restrictions on freedom of movement. Domain prevalence differed sharply by trafficking classification, indicating that these domains capture meaningful variation in exploitation experiences. However, when all domains were modeled simultaneously, and the models additionally accounted for economic strain, food security, and self-efficacy, no single domain showed a statistically significant unique association with suicidality. Restriction of movement and surveillance showed the most consistent trend toward higher odds of any suicidality, suggesting that constraint-related experiences may warrant attention as a potentially relevant marker of risk, even when effects are modest and not statistically significant.

Notably, a cumulative exposure measure supported a modest dose–response association with suicidality severity, indicating that the accumulation of trafficking-related harms may be more closely aligned with suicidality intensity than any single domain considered in isolation. This cumulative pattern was less evident for the binary suicidality outcome, consistent with reduced sensitivity of dichotomized measures. Taken together, these findings suggest that screening and referral efforts may be improved by capturing cumulative trafficking-related harms and indicators of restricted freedom, rather than relying solely on a binary trafficking classification. This interpretation aligns with guidance that emphasizes restricted freedom, document confiscation, and coercion-related constraints as key indicators for identifying and referring individuals for services (30, 35, 36).

Findings suggest that suicidality among women engaged in commercial sex in this setting was more closely associated with material hardship and perceived coping capacity than with binary sex trafficking classification alone. Programmatically, responses for women engaged in commercial sex and those classified as sex trafficking may benefit from integrating economic strengthening supports, such as addressing material deprivation and food insecurity, with psychosocial components that strengthen coping capacity and self-efficacy. The domain findings also suggest that screening and referral protocols should move beyond a binary trafficking classification and include assessment of cumulative trafficking-related harms and indicators of restricted freedom, such as mobility restriction, surveillance, and document or phone confiscation. Because the domain-specific associations were modest and not statistically definitive, these implications should be interpreted as practice-oriented guidance for more comprehensive screening and service planning.

### 5.2 Limitations

Several limitations should be considered. First, the data are cross-sectional, so temporal ordering and causal pathways cannot be established. Second, some trafficking indicators displayed skip-pattern missingness; however, conclusions remained robust to alternative domain constructions that excluded the highest-missingness items. Third, mixture or latent class estimation incorporating RDS weights was not conducted; instead, weights were applied in descriptive prevalence and regression models, consistent with the study’s focus on parsimonious and interpretable inference. Finally, suicidality and coping were measured by self-report, and food security was assessed with a single item, which may attenuate associations.

### 5.3 Future research

There is need to document the processes through which women enter commercial sex and how they may experience or be at risk for experiencing sex trafficking. The distinction between commercial sex and sex trafficking is perhaps clearer in developing countries, where commercial sex is legal and regulated for adults. In such settings, it is feasible to uphold the rights of women engaged in commercial sex and to ensure minimum standards for their working conditions. This is not the case in less developed contexts, and especially in the outer posts within these countries, where the conditions for work are virtually similar for those in legal commercial sex and those who are sex trafficking victims. Stated otherwise, there are contexts where legalization of commercial sex for women may not result in significant differences for their outcomes, and interventions should not be narrowly focused on those identified only as experiencing sex trafficking.

Economic empowerment programs focusing on building the financial capability of survivors and those at risk of human trafficking (3, 38, 39) could result in several proximal outcomes that include knowledge of local economy or gaining business and alternative job skills for those who wish to pursue other professions as we found in our context (3) and distal outcomes such as economic independence and the ability to make basic needs for this population. This, in turn, would reduce the risks of trafficking for the purposes of sexual exploitation for many younger women who are economically vulnerable, and where trafficking rings exist.

## 6 Conclusion

The purpose of this study was to examine whether suicidality and related economic and coping indicators differ between women engaged in commercial sex who were classified as having experienced sex trafficking versus those not classified as having been trafficked, and to identify correlates of suicidality in this population. In the full analytic sample, survey-weighted multivariable models showed a consistent pattern: greater economic strain was associated with higher suicidality, whereas higher self-efficacy was strongly protective, even after adjusting for age and sex trafficking classification. Food security showed a smaller, trend-level protective association with suicidality in these adjusted models. In contrast, sex trafficking classification itself was not independently associated with suicidality once economic strain, food security, self-efficacy, and age were included, suggesting that proximate economic hardship and perceived coping capacity were more strongly related to suicidality than the binary trafficking classification.

Sex trafficking victimization and engagement in commercial sex show no differences in suicidal ideation among women in our sample. This could be because the conditions that the two groups work in are virtually similar. Given the modest differences identified in this study, service providers who are focused on providing services solely to victims of sex trafficking should be mindful that others may also need intervention, especially those who are engaged in commercial sex in developing countries. Several organizations are focused on psychosocial interventions. Addressing the physical and economic needs of survivors can enhance their psychological well-being, given that economic strain was associated with higher suicidality severity, even after adjusting for age, economic strain, food security, and self-efficacy.

## Data Availability

The raw data supporting the conclusions of this article will be made available by the authors, without undue reservation.
